# A comparison of placebo and nocebo effects on objective and subjective postural stability: a double-edged sword?

**DOI:** 10.3389/fnhum.2022.967722

**Published:** 2022-08-18

**Authors:** Katherine Russell, Michael Duncan, Michael Price, Amber Mosewich, Toby Ellmers, Mathew Hill

**Affiliations:** ^1^Centre for Sport, Exercise and Life Sciences, School of Life Sciences, Coventry University, Coventry, United Kingdom; ^2^Faculty of Kinesiology, Sport, and Recreation, University of Alberta, Edmonton, AB, Canada; ^3^Department of Brain Sciences, Imperial College London, London, United Kingdom

**Keywords:** placebo, nocebo, expectation, postural control, subjective stability, belief

## Abstract

**Background:** Positive expectations (i.e., placebo effect) can improve postural control during quiet standing. This raises an important question: if postural control is susceptible to positive expectations, is it possible to elicit the opposite, a decline in postural stability, simply by suggesting a performance impairment (i.e., nocebo) will take place? Yet no studies have examined the nocebo effect on balance performance. To better understand both phenomena, comparative studies, which include both placebo and nocebo conditions, are needed.

**Method:** Forty-two healthy adults were initially assessed for objective (center of pressure movement) and subjective (perceived) postural stability and performance expectations. Participants were then randomly assigned in equal numbers to a placebo (positive expectation), nocebo (negative expectation) or control (no suggestion) group. Participants in the placebo/nocebo groups were deceptively administered an inert capsule described as a potent supplement which would either positively or negatively influence their balance performance. Objective and subjective postural stability, and performance expectations were reassessed 20 min later.

**Results:** The nocebo procedure evoked an increase in COP sway movements and reduced perceived stability compared to a control group. The placebo group presented with reductions COP sway movements and increased perceived stability following expectation manipulation. Compared to the control group, the placebo group showed a significantly higher performance expectation whilst the nocebo group showed a significantly lower performance expectation. Regression analyses also revealed that performance expectations following the placebo/nocebo procedure significantly predicted perceptions of postural instability (i.e., perceived performance), accounting for around 50% of the variance. These results remained even when controlling for *actual* performance (i.e., objective postural stability).

**Conclusion:** Our findings indicate that positive and negative performance expectations evoked by instructional manipulation can profoundly influence both objective and subjective postural stability. Postural control—and perceptions regarding such—are clearly susceptible to expectation manipulation, which could have important practical implications and repercussions on testing, training interventions and rehabilitation programs. Positive and negative expectancies are a double-edged sword for postural control.

## Introduction

Placebos and nocebos are physiologically inert substances (i.e., pharmacological/nutritional) or sham interventions (i.e., psychological, physical or mechanical), which produce complex psychobiological responses independent of any direct therapeutic effects (Price et al., 2008; Turi et al., 2018). The *placebo* effect (originating from the Latin phrase “I will please”) refers to a desirable outcome attributable to a purported beneficial treatment (Hurst et al., 2020). In contrast, the *nocebo* effect (originating from the Latin phrase “I will harm”) refers to an undesirable outcome to a purported harmful treatment, administered with or without deliberate damage intention (Beedie et al., 2018). Placebo and nocebo effects are often explained on the basis of the recipient’s expectancies about the received substance or intervention. These expectancies may be the result of conscious (e.g., expectations) and non-conscious (e.g., conditioning) cognitive or affective processes (Wager and Atlas, 2015).

Although placebo (analgesia) and nocebo (hyperalgesia) phenomena have been largely confined to the study of pain tolerance (Frisaldi et al., 2015), there is emerging evidence elucidating the application of these effects on motor and cognitive performance. In this regard, it is now well established that placebo effects can positively influence muscle force production (Fiorio et al., 2014; Villa-Sánchez et al., 2019), increase fatigue resistance (Piedimonte et al., 2015) and improve attention/vigilance (Colagiuri and Boakes, 2010). Despite convincing evidence that placebo related expectations can induce positive changes in several cognitive/motor functions, the potential effects of placebos on other important cognitive-motor functions that are essential to normal everyday functioning, such as balance performance, are less well understood.

It is well established that older adults frequently hold inappropriate expectations related to their balance abilities, with many individuals holding either over- or under-confident beliefs (Delbaere et al., 2010). Despite this, there has been little focus in the literature on how such performance expectations affect balance, as well as the efficacy of procedures (e.g., placebos) that can be used to modify these beliefs. Indeed, only one study has examined changes in postural control following a placebo procedure. Young adults who were made to believe that a placebo treatment was effective (application of an inert electrical device over the leg muscle) presented with reduced postural sway and perceived their balance control to be subsequently more stable when compared to a control group (Villa-Sánchez et al., 2019). These findings indicate that instead of being regarded as a bias to control for in randomized control trials, placebos could be deliberately utilized and combined with established approaches to increase therapeutic efficacy of balance interventions (Enck et al., 2013; Schwarz and Büchel, 2015). Although the initial work of Villa-Sánchez and colleagues offers valuable insight into the effects of placebo effects on objective and subjective balance performance, to our knowledge, no studies have examined the nocebo effect on balance performance. In order to better understand both phenomena, comparative studies, which include both placebo and nocebo conditions, are needed.

Previous research has shown that information disclosure about potential side effects of a treatment (i.e., nocebo), may create expectancies which contribute to adverse effects and prevention of improvement. For example, the nocebo effect can negatively influence muscle force production (Pollo et al., 2012; Emadi Andani et al., 2015; Corsi et al., 2019), endurance performance (McLemore et al., 2020), vigilance (Harrell and Juliano, 2009), response accuracy (Turi et al., 2018), and reaction time (Colagiuri et al., 2011). However, the interaction between balance performance and the nocebo effect are unknown. This raises an important question: if postural control is susceptible to positive expectations, is it possible to elicit the opposite, a decline in postural stability, simply by suggesting a performance impairment will take place? If so, this would have far-reaching implications for applied practice, given that such negative expectations could potentially elicit profound repercussions by interfering with training adaptations. Nocebos have also been shown to affect perceptual processes, resulting in individuals perceiving stimuli as being more painful and fatiguing (Reicherts et al., 2016; Wolters et al., 2019; Feldhaus et al., 2021). Given the clear dissociation between *actual* and *perceived* postural instability in a range of clinical balance disorders (Kaski, 2020; Castro et al., 2022), is it possible to induce such similarly distorted perceptions of instability *via* negative performance expectations elicited *via* a nocebo? Research is needed to fill these knowledge gaps.

The aim of the present study is to directly compare the influence of placebo and nocebo instructions on objective and subjective postural stability and performance expectancies compared to a no-treatment control group. The utilization of a no-treatment control will enable us to accurately estimate the relative magnitude of placebo and nocebo effects in response to treatments. Our hypotheses are as follows: (1) performance expectations would be high in the placebo group and low in the nocebo group; (2) positive performance expectancies (placebo) would result in improved objective (i.e., center of pressure movements) and subjective (i.e., perceived stability) postural stability; and (3) negative performance expectancies (nocebo) would result in reduced objective and subjective postural stability. Finally, we also predicted that performance expectations would predict perceptions of stability (i.e., perceived performance) irrespective of *actual* stability (i.e., objective performance).

## Methods

### Participants

Effect size (Cohen’s *d*) were calculated from a similar study from mean changes in postural sway (large effect size, *d* = 1.20) in a placebo group (Villa-Sánchez et al., 2019). Power analysis (G*Power, v3.1.9.4) showed that for a repeated measures ANOVA analyses a minimum of 42 participants (*n* = 14 per group) would be required to be able to detect a significant within-between interaction of medium effect size [assuming 1-*β* = 80%, *α* = 0.05, Cohen’s *f* = 0.25 (standardized medium effect size), three groups, and two within-subject conditions]. Whilst previous research has reported a large effect size of placebo effects on balance performance (Villa-Sánchez et al., 2019), we chose a more conservative medium effect size estimate because the relatively low number of investigations will inherently increase the uncertainty of the true population estimate. All participants initially completed a health screening questionnaire to assess eligibility for the study. Inclusion criteria were age between 18 and 35 years. Exclusion criteria were self-reported history of psychiatric, neurological, cardiovascular or pulmonary diseases, orthopedic pathology or musculoskeletal dysfunctions. Additionally, none of the participants reported any allergies, prior adverse responses to medication, or current health problems requiring medication were excluded from the study to minimize the possibility of adverse responses to the belief that an active treatment had been received (Beedie, 2007). Following baseline assessment, all participants were randomly assigned to one of three groups: (1) placebo (positive belief); (2) nocebo (negative belief); and (3) control (no suggestion; Table 1). There were no statistically significant differences between the three groups for age, sex or self-reported physical activity levels (*p* > 0.05). Participants provided written, informed consent prior to data collection. The experimental procedures were carried out in accordance with the standards outlined in the declaration of Helsinki (1964) and the study received approval by the institutional ethics committee (Application ID: P126096).

**Table 1:** Mean and SD participant characteristics.

	**Placebo**	**Nocebo**	**Control**
	**(*n* = 14)**	**(*n* = 14)**	**(*n* = 14)**
Sex (female)	7	6	7
Age (years)	21.0 ± 1.7	20.1 ± 0.9	23.0 ± 3.3
Height (m)	1.80 ± 0.09	1.76 ± 0.08	1.79 ± 0.08
Mass (kg)	74.3 ± 13.1	74.2 ± 15.9	75.3 ± 16.3
BMI (kg/m^2^)	22.9 ± 2.2	23.8 ± 4.3	24.7 ± 4.1
Physical activity (h·wk^−1^)	2.6 ± 1.2	2.6 ± 0.9	2.7 ± 1.4

### Design

**Figure 1:**
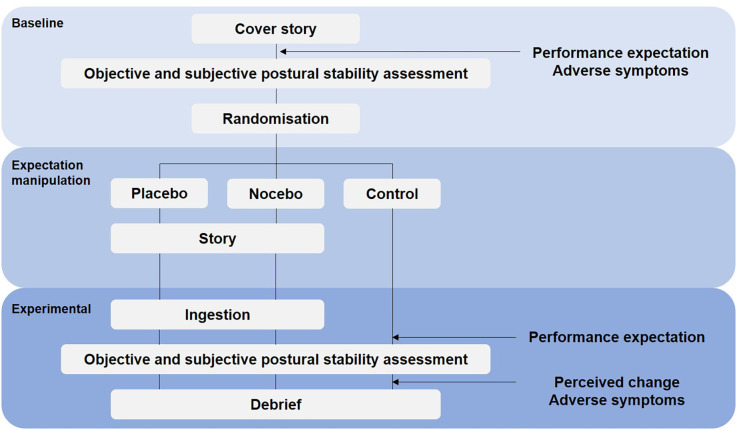
Schematic of the experimental design.

To minimize cross-contamination between experimental and control treatments, this study was conducted as a randomized controlled parallel trial (Figure 1). The importance of a no-treatment control group alongside placebo and/or nocebo group has recently been highlighted in the literature (Beedie et al., 2018; Colloca and Barsky, 2020). Primary outcome measures were objective (posturography) and subjective (perceived postural stability) balance performance. Secondary outcomes were subjective performance expectation, perceived change in performance, and adverse symptoms. Following baseline assessments, participants were allocated to a placebo, nocebo or control group. The randomization process was done using Research Randomizer, a program published on a publicly accessible official website^1^. During baseline assessments, the principle investigator was blind to treatment allocation. Objective and subjective postural stability and performance expectations were reassessed 20 min later. Subjective performance expectation was measured before baseline and experimental conditions. Perceived change in performance was rated after the experimental trial. Adverse symptoms were assessed before the baseline condition and after the experimental trial. In accordance with recent recommendation (Horváth et al., 2021) participants in the no-treatment control group underwent the same procedure but did not receive any capsules and were not further instructed regarding positive or negative suggestions (Figure 1).

### Belief manipulation

In accordance with previous experiments (i.e., Beedie, 2007; Hurst et al., 2017), during the 20-min interval between baseline and experimental conditions, participants in the placebo and nocebo groups were administered a capsule described as a potent sports supplement, “inorganic nitrate”. Participants in the placebo group (i.e., positive belief treatment) were asked to ingest two, size 0 (volume; 0.68 ml, size; 21.5 mm) clear gelatine capsules containing 200 mg of corn flour (Morrison’s, Bradford, UK) and informed that inorganic nitrate would “improve mental alertness and enhance muscle force production”, which are important determinants of balance performance. Participants in the nocebo group (i.e., negative belief treatment) were also asked to ingest two, size 0 (volume; 0.68 ml, size; 21.5 mm) clear gelatine capsules containing 200 mg of corn flour, but were informed that inorganic nitrate can “dampen the activity of the central nervous system, reduce alertness and causes sensations of tiredness, fatigue and lethargy” which would decrease balance performance. Similar to previous studies (Hurst et al., 2017), the effectiveness of the belief manipulation (placebo and nocebo groups) was assessed during a debrief immediately after the experimental trials, at which point the true nature of the study was revealed. Participants were asked to respond on a visual analogue scale (VAS) “how much did you believe the treatment influenced your performance” (from 0, no influence to 10, high influence). We also asked participants to respond on a VAS, “how much did you believe the information you received” (from 0, did not believe at all to 10, completely believed).

### Postural stability

#### Quantitative posturography

During baseline and experimental assessments, participants performed three 30-s quiet standing trials on a force platform (AMTI, AccuGait, Watertown, MA) with 15-s rest between each trial. To further explore the possible mediating influence of task difficulty, participants completed trials under both bipedal and unipedal conditions, in a randomized order. To ensure continuity between trials, participants were unshod and instructed to stand quietly with the hands clasped together in front of the body. In the bipedal stance, participants stood with the feet together (Romberg stance; Objero et al., 2019). For the unipedal trials, participants maintained a single-leg stance with the dominant limb (defined as the foot used to kick a ball). Participants were instructed that the unloaded leg should not touch the supporting leg and the knee should be flexed to 90°. During all quiet standing trials, participants were asked stand quietly on the force platform while and minimizing any extraneous movements and gazing at a target 1.5 m from the force platform. Participants could *step off* the plate and rest between trials (±15 s). Ground reaction force data were sampled at 100 Hz (AMTI, Netforce, Watertown, MA) and filtered using a fourth-order low-pass (6 Hz) Butterworth filter (BioAnalysis, V2.2, AMTI) prior to calculation of center of pressure (COP) metrics. The maximal displacement (i.e., distance between the maximum and minimum COP displacement) of center of pressure (COP) in the anteroposterior (AP) and mediolateral (ML) directions (both cm) were subsequently calculated (AMTI, BioAnalysis, Version 2.2, Watertown, MA). The validity and reliability of these parameters have previously been established for this sampling duration (Pinsault and Vuillerme, 2009). An average of the three trial trials (total 90 s) recorded during baseline and experimental conditions were used in the subsequent analyses (Ruhe et al., 2010).

#### Perceived postural (in)stability

Immediately following each 30-s quiet standing trial, participants were asked to rate their degree of instability during the trial (“how stable did you feel during the trial?”) using a 0–10 VAS, where 0 corresponded to being “completely steady” and 10 “so unsteady that I would fall” (Castro et al., 2019). This was based on the subjective stability scoring system originally proposed by Schieppati et al. (1999) and has been shown to be valid and reliable for bipedal and unipedal balance tasks in healthy young adults (Hauck et al., 2008). As with posturographic data, an average of the three trials recorded during baseline and experimental conditions were used in subsequent analyses.

### Questionnaires and self-report

#### Subjective performance expectation

To assess participants subjective performance expectation (also referred to as self-efficacy), we used the item “I will perform well in the task” (Winkler and Hermann, 2019) to be rated on a VAS ranging from 0 to 10 (0 being “do not agree at all”, 5 “neutral” and 10 being “totally agree”). Performance expectation was measured before each block of baseline and experimental trials (Figure 1).

#### Perceived change in performance

Immediately following the experimental trials, participants were asked to rate the perceived change in balance performance between the baseline condition (i.e., before performance expectation manipulation) and the experimental assessment (i.e., after performance expectation manipulation; Figure 1). Participants were asked “how do you rate your balance performance now in comparison to the first assessment?” on a VAS ranging from 0 (worse) to 10 (better).

#### Adverse symptoms

Perceived adverse symptoms (or side effects) of the administered capsules were assessing using an adapted version of the Generic Assessment of Side Effects Scale (GASE; Rief et al., 2011). Although the original GASE consists of 36 symptoms, for the purpose of our study, 12 adverse symptoms were selected to match the potential side effects described in the participant information sheet (headache, fatigue, irritability, dizziness, nausea, feeling of weakness, drowsiness, tremor, muscle pain, anxiety/fear). As recommended (Rheker et al., 2018; Winkler and Hermann, 2019), we assessed adverse symptoms before the baseline and after the experimental conditions.

### Statistical analysis

Data were analyzed using SPSS version 25.0 (IBM Inc., Chicago, IL). For all analyses, normality (Shapiro–Wilk Test) and homogeneity of variance/sphericity (Mauchly Test) were performed and confirmed prior to parametric analyses. To examine differences in objective and subjective postural stability, subjective performance expectation and adverse symptoms, a series of two-way mixed model analysis of variance (ANOVA) were undertaken (with Bonferroni correction) to test for the within-subject effects of time [× 2 (baseline vs. experimental)] and between subject effects of group [× 3 (placebo vs. nocebo vs. control)]. Therefore, where VAS data was normally distributed, parametric tests were employed. A one-way ANOVA was undertaken to assess differences in perceived change in performance between the three groups (placebo vs. nocebo vs. control). The effectiveness of belief manipulation was assessed using an independent t-test (placebo vs. nocebo). The Mann-Whitney *U* test was used for adverse symptoms (GASE: 0 = no symptoms, 1 = mild symptoms, 2 = moderate symptoms, 3 = severe symptoms) for pairwise comparisons. Where significant interactions or main effects were detected, *post hoc* analyses using Bonferroni-adjusted *α* determined the location of any differences. For ANOVA, effect sizes are reported as partial eta-squared value (*η*^2^). Cohen’s *d* effect sizes (ES) are reported for *post hoc* comparisons with an effect size of 0.2, 0.6, 1.2 and 2.0 indicating small, medium, large and very large effects, respectively. The alpha value was *a priori* set at *p* < 0.05 for all tests. Given that placebo effects can demonstrate considerable variability (Beedie and Foad, 2009), we reported inter-individual responses to treatments.

We also performed regressional analyses to examine whether performance expectations influence perceptions of postural stability (i.e., perceived performance) independent of *actual* stability (i.e., objective performance). We conducted a separate regression for each task (bipedal vs unipedal) and condition (pre- and post-belief manipulation). For each regression, perceived stability for that given task/condition was entered as the outcome variable, whilst the predictor variables were: performance expectation (for that given condition) and objective task performance for that given task/condition (AP and ML COP displacement). The assumptions of homoscedasticity (inspecting the standardized residuals by standardized predicted values plot), error-independence (Durbin–Watson values = 1.67–1.83), lack of multicollinearity (variance inflation factors <2.05, tolerances >0.49), and normal distribution of errors (determined with Kolmogorov–Smirnov tests and inspection of histogram of residuals) were verified.

## Results

### Performance expectations

**Figure 2:**
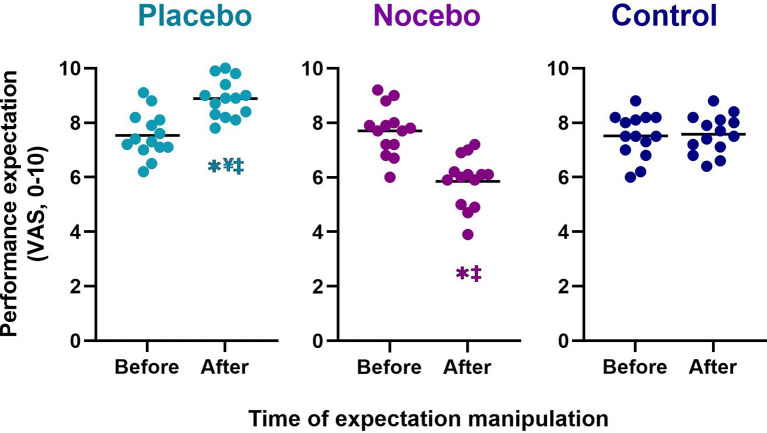
Performance expectation before the first balance assessment and after the expectation manipulation for placebo, nocebo, and control groups. *Statistically significantly different to before the first balance assessment. ^¥^Statistically significantly different to nocebo group after expectation manipulation. ^‡^Statistically significantly different to control group after expectation manipulation.

The mixed-model ANOVA revealed a statistically significant group × time interaction for performance expectation (*F*_(2,78)_ = 27.462, *p* < 0.001, $\eta_{p}^{2}$ = 0.413). Follow up *post hoc* analyses revealed that the groups differed significantly in their performance expectations after expectation manipulation, but there were no differences at baseline (Figure 2). Participants in the placebo group (*M* = 8.9, *SD* = 0.7) reported a statistically significantly higher performance expectation than participants in the nocebo (*M* = 5.9, *SD* = 0.9, *p* < 0.001, *M*_diff_ = 3.02, *d* = 3.71) and control (*M* = 7.6, *SD* = 0.7, *p* < 0.001, *M*_diff_ = 1.29, *d* = 1.88) group, after expectation manipulation. The nocebo group reported a statistically significantly lower performance expectation that the control group (*p* < 0.001, *M*_diff_ = 1.73, *d* = 2.09), after expectation manipulation. Moreover, performance expectation increased significantly in the placebo group following the expectation manipulation (*p* < 0.001*, M*_diff_ = 1.34, *d* = 1.79), and it decreased significantly in the nocebo group (*p* < 0.001*, M*_diff_ = 1.86, *d* = 2.03) but not the control group (*p* > 0.05, *M*_diff_ = 0.06, *d* = 0.08).

### Perceived change in performance and effectiveness of belief manipulation

**Figure 3:**
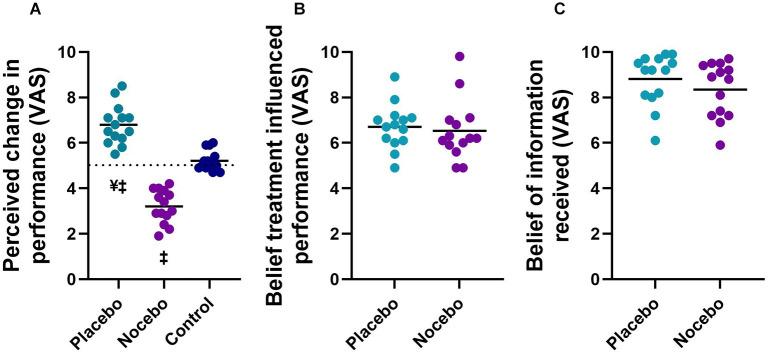
Perceived change in balance performance **(A)**, the degree to which participants believed the treatment influenced their performance **(B)**, and the degree to which participants believed the information they received **(C)** in placebo, nocebo, and control groups. ^¥^Statistically significantly different to nocebo group. ^‡^Statistically significantly different to control group after expectation manipulation.

The mixed-model ANOVA revealed a statistically significant group main effect (*F*_(2,41)_ = 92.248, *p* < 0.001) for perceived change in balance performance (Figure 3A). Follow up *post hoc* analyses revealed that the placebo group (*M* = 6.8, *SD* = 0.9) reported a statistically significantly greater improvement in performance than the nocebo (*M* = 3.2, *SD* = 0.7, *p* < 0.001, *M*_diff_ = 3.6, *d* = 4.48) and control group (*M* = 5.2, *SD* = 0.4, *p* < 0.001, *M*_diff_ = 1.6, *d* = 2.31), whilst the nocebo group reported a statistically significantly lower balance performance compared to the control group (*p* < 0.001, *M*_diff_ = 2.0, *d* = 3.34). There was no statistically significant difference in perceived belief that the treatment influenced participants performance between the placebo (*M* = 6.7, *SD* = 1.0) and nocebo (*M* = 6.5, *SD* = 1.3, *p* > 0.05, *M*_diff_ = 0.2, *d* = 0.18) groups (Figure 3B). All participants in the placebo (*M* = 8.8, *SD* = 1.1) and nocebo (*M* = 8.3, *SD* = 1.2) group believed the information that they received (scored > than 5; neutral; Figure 3C). There was no difference in how much participants believed the information that they received between the placebo and nocebo groups (*p* > 0.05, *M*_diff_ = 0.5, *d* = 0.40).

### Objective and subjective postural stability during bipedal stance

**Figure 4:**
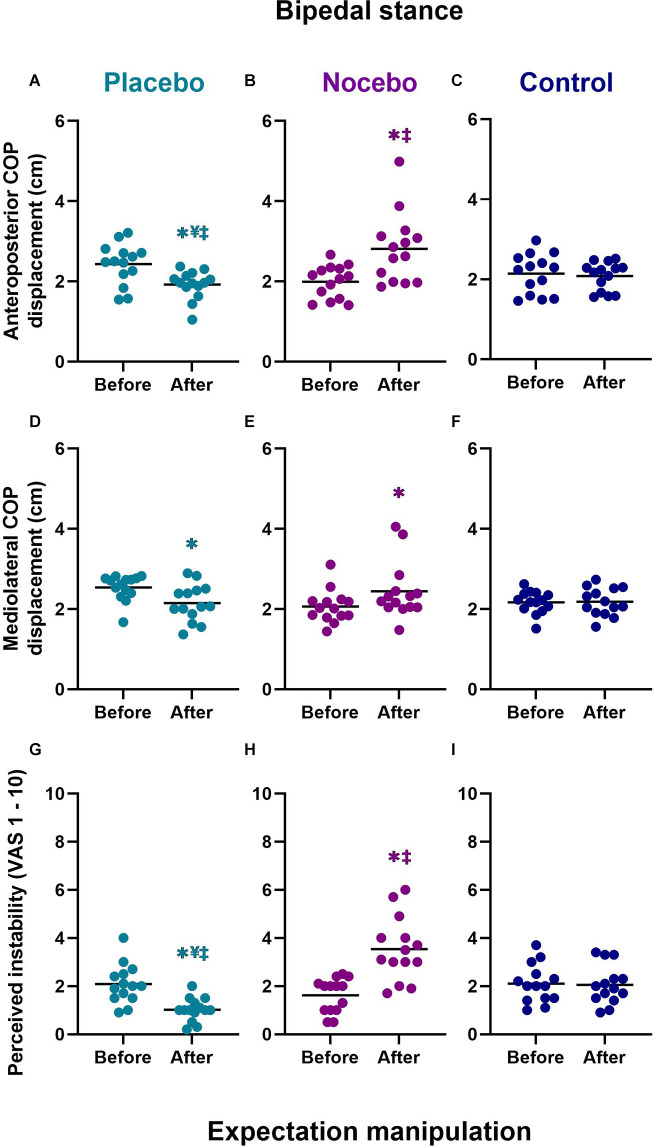
Objective **(A–F)** and subjective **(G–I)** bipedal balance performance before and after expectation manipulation for placebo, nocebo, and control groups. *Statistically significantly different to before the first balance assessment. ^¥^Statistically significantly different to nocebo group after expectation manipulation. ^‡^Statistically significantly different to control group after expectation manipulation.

Mixed-model ANOVA revealed a statistically significant group × time interaction for the anteroposterior COP range (*F*_(2,78)_ = 11.471, *p* < 0.001, $\eta_{p}^{2}$ = 0.227), mediolateral COP range (*F*_(2,78)_ = 5.338, *p* = 0.007, $\eta_{p}^{2}$ = 0.120) and subjective postural stability (*F*_(2,78)_ = 21.780, *p* < 0.001, $\eta_{p}^{2}$ = 0.358; Figure 4).

#### Anteriorposterior COP range

Although there were no differences at baseline, participants in the placebo group (*M* = 1.92, *SD* = 0.35) demonstrated a statistically significantly smaller anteroposterior COP range than participants in the nocebo (*M* = 2.81, *SD* = 0.87, *p* < 0.001, *M*_diff_ = 0.89, *d* = 1.35) but not the control (*M* = 2.08, *SD* = 0.35, *p* > 0.05, *M*_diff_ = 0.16, *d* = 0.46) group, after expectation manipulation (Figures 4A–C). The nocebo group reported a statistically significantly greater anteroposterior COP range than the control group (*p* = 0.001, *M*_diff_ = 0.73, *d* = 1.11), after expectation manipulation. Moreover, anteroposterior COP range decreased significantly in the placebo group (*p = 0*.012*, M*_diff_ = 0.51, *d* = 1.16), and it increased significantly in the nocebo group (*p* < 0.001*, M*_diff_ = 0.82, *d* = 1.21) but not the control group (*p* > 0.05, *M*_diff_ = 0.06, *d* = 0.14), with respect to baseline assessments.

#### Mediolateral COP range

After expectation manipulation, there were no statistically significant differences in the mediolateral COP range between the placebo (*M* = 2.15, *SD* = 0.46) with nocebo (*M* = 2.44, *SD* = 0.71, *M*_diff_ = 0.30, *d* = 0.50) or control (*M* = 2.18, *SD* = 0.35, *M*_diff_ = 0.04, *d* = 0.09) groups, or between the nocebo and control (*M*_diff_ = 0.26, *d* = 0.47) groups (all *p* > 0.05; Figures 4D–F). However, the mediolateral COP range decreased significantly in the placebo group (*p* = 0.021*, M*_diff_ = 0.39, *d* = 1.0), and it increased significantly in the nocebo group (*p = 0*.026*, M*_diff_ = 0.38, *d* = 0.65) but not the control group (*p* > 0.05, *M*_diff_ = 0.01, *d* = 0.04), with respect to baseline assessments.

#### Subjective postural (in)stability

Although there were no differences at baseline, participants in the placebo group (*M* = 1.02, *SD* = 0.48) reported statistically significantly lower subjective instability than participants in the nocebo (*M* = 3.54, *SD* = 1.32, *p* < 0.001, *M*_diff_ = 2.51, *d* = 2.54) and the control (*M* = 2.06, *SD* = 0.81, *p* = 0.006 *M*_diff_ = 1.04, *d* = 1.56) group, after expectation manipulation (Figures 4G–I). The nocebo group reported a statistically significantly greater subjective instability than the control group (*p* < 0.001, *M*_diff_ = 1.48, *d* = 1.35), after expectation manipulation. Moreover, subjective postural instability decreased significantly in the placebo group (*p* = 0.002*, M*_diff_ = 1.06, *d* = 1.59), and it increased significantly in the nocebo group (*p* < 0.001*, M*_diff_ = 1.91, *d* = 1.81) but not the control group (*p* > 0.05, *M*_diff_ = 0.04, *d* = 0.05), with respect to baseline assessments.

### Objective and subjective postural stability during unipedal stance

**Figure 5:**
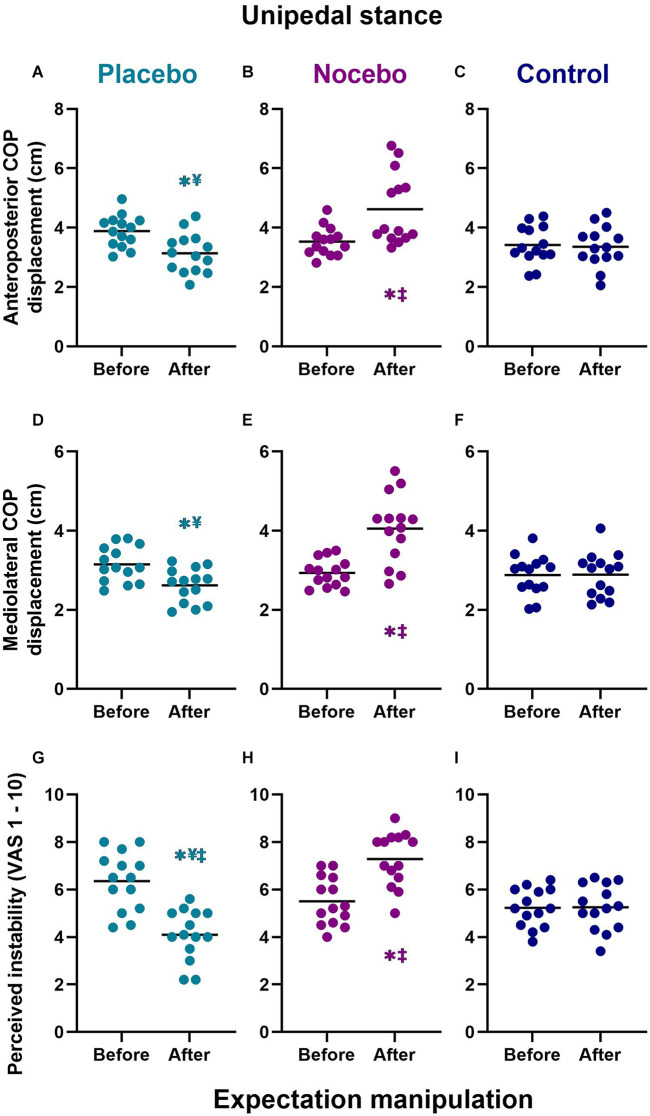
Objective **(A–F)** and subjective **(G–I)** unipedal balance performance before and after expectation manipulation for placebo, nocebo, and control groups. *Statistically significantly different to before the first balance assessment. ^¥^Statistically significantly different to nocebo group after expectation manipulation. ^‡^Statistically significantly different to control group after expectation manipulation.

For unipedal stance, the mixed-model ANOVA revealed a statistically significant group × time interaction for the anteroposterior COP range (*F*_(2,78)_ = 14.244, *p* < 0.001, $\eta_{p}^{2}$ = 0.268), mediolateral COP range (*F*_(2,78)_ = 13.613, *p* < 0.001, $\eta_{p}^{2}$ = 0.259) and perceived postural stability (*F*_(2,78)_ = 26.553, *p* < 0.001, $\eta_{p}^{2}$ = 0.405; Figure 5).

#### Anteriorposterior COP range

Although there were no differences at baseline, participants in the placebo group (*M* = 3.14, *SD* = 0.67) demonstrated a statistically significantly smaller anteroposterior COP range than participants in the nocebo (*M* = 4.48, *SD* = 1.25, *p* < 0.001, *M*_diff_ = 1.34, *d* = 1.34) but not the control (*M* = 3.36, *SD* = 0.68, *p* > 0.05 *M*_diff_ = 0.22, *d* = 0.33) group, after expectation manipulation (Figures 5A–C). The nocebo group reported a statistically significantly greater anteroposterior COP range the control group (*p* = 0.001, *M*_diff_ = 1.12, *d* = 1.11), after expectation manipulation. Moreover, anteroposterior COP range decreased significantly in the placebo group (*p* = 0.011*, M*_diff_ = 0.75, *d* = 1.24), and it increased significantly in the nocebo group (*p* < 0.001*, M*_diff_ = 1.38, *d* = 1.43) but not the control group (*p* > 0.05, *M*_diff_ = 0.06, *d* = 0.09), with respect to baseline assessments.

#### Mediolateral COP range

Although there were no differences at baseline, participants in the placebo group (*M* = 2.62, *SD* = 0.43) demonstrated a statistically significantly smaller mediolateral COP range than participants in the nocebo (*M* = 4.05, *SD* = 0.86, *p* < 0.001, *M*_diff_ = 1.44, *d* = 2.11) but not the control (*M* = 2.89, *SD* = 0.55, *p* > 0.05 *M*_diff_ = 0.27, *d* = 0.55) group, after expectation manipulation (Figures 5D–F). The nocebo group reported a statistically significantly greater mediolateral COP range than the control group (*p < 0*.001, *M*_diff_ = 1.16, *d* = 1.61), after expectation manipulation. Moreover, mediolateral COP range increased significantly in the nocebo group (*p* < 0.001*, M*_diff_ = 1.12, *d* = 1.71) but not the placebo (*p* > 0.05, *M*_diff_ = 0.53, *d* = 1.21) or control group (*p* > 0.05, *M*_diff_ = 0.01, *d* = 0.02), with respect to baseline assessments.

#### Subjective postural (in)stability

Although there were no differences at baseline, participants in the placebo group (*M* = 4.09, *SD* = 1.07) demonstrated a statistically significantly lower subjective postural instability than participants in the nocebo (*M* = 7.29 *SD* = 1.13, *p* < 0.001, *M*_diff_ = 3.19, *d* = 2.90) and the control (*M* = 5.25, *SD* = 0.96, *p* = 0.013 *M*_diff_ = 1.16, *d* = 1.14) group, after expectation manipulation (Figures 5G–I). The nocebo group reported a statistically significantly greater subjective instability than the control group (*p* < 0.001, *M*_diff_ = 2.04, *d* = 1.94), after expectation manipulation. Moreover, subjective postural instability decreased significantly in the placebo group (*p* < 0.001*, M*_diff_ = 2.26, *d* = 1.97), and increased significantly in the nocebo group (*p* < 0.001*, M*_diff_ = 1.79, *d* = 1.66) but not the control group (*p* > 0.05, *M*_diff_ = 0.02, *d* = 0.02), with respect to baseline assessments.

### Adverse symptoms

**Figure 6:**
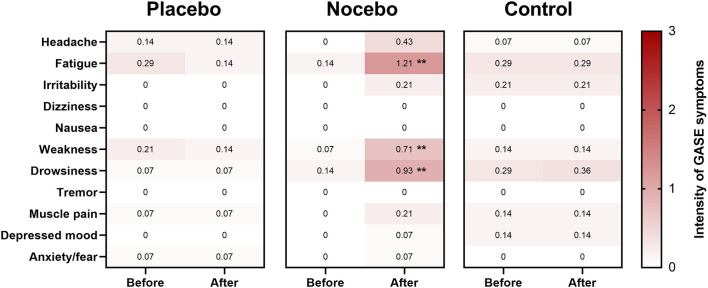
Heat map of adverse symptoms (or side effects) for the placebo, nocebo and control group. Shading indicates strength of the adverse effect [white = 0 (no symptoms), light pink = 1 (mild symptoms), dark pink = 2 (moderate symptoms), 3 = red (severe symptoms)]. Value in each box represents the mean response. **Statistically significantly different to both placebo and control groups (*p* < 0.05; adjusted by Bonferroni correction).

Mann-Whitney *U* test showed a statistically significant difference in the level of perceived fatigue reported in the nocebo group compared to the placebo (*U* = 23.0, *p* < 0.001) and control (*U* = 32.0, *p* = 0.001) group after expectation manipulation (Figure 6). Similarly, there was statistically significant difference in the level of perceived weakness reported in the nocebo group compared to the placebo (*U* = 32.5, *p* = 0.001) and control (*U* = 55.5, *p* = 0.031) group after expectation manipulation (Figure 6). Finally, there was a statistically significant difference in the level of perceived drowsiness reported in the nocebo group compared to the placebo and control (both; *U* = 60.0, *p* = 0.034) group after expectation manipulation (Figure 6).

### Regression analyses

The regression models did not significantly predict perceived instability during the pre-manipulation phase, for either bipedal (*R*^2^ = 0.079, *F*_(3,38)_ = 1.08, *p* = 0.369) or unipedal task performance (*R*^2^ = 0.055, *F*_(3,38)_ = 0.73, *p* = 0.539). However, post-manipulation, the regression model for both bipedal (*R*^2^ = 0.577, *F*_(3,38)_ = 17.30, *p* < 0.001) and unipedal (*R*^2^ = 0.560, *F*_(3,38)_ = 16.13, *p* < 0.001) task performance was significant. In both models, the only significant predictor of perceived instability was performance expectations (bipedal: *β* = −0.73, *p* < 0.001, unipedal: *β* = −0.57, *p* = 0.003); with lower performance expectations predicting greater perceived instability. In contrast, neither *actual* COP displacement in either the AP (bipedal: *β* = −0.05, *p* = 0.860, unipedal: *β* = 0.30, *p* = 0.175) or ML direction (bipedal: *β* = −0.03, *p* = 0.926, unipedal: *β* = 0.31, *p* = 0.272) significantly predicted perceptions of instability.

## Discussion

In this study, we investigated whether placebo and nocebo effects in postural stability can be elicited by evoking positive and negative expectations about task performance. Performance expectations were manipulated by deceptively instructing participants about alleged beneficial (placebo group) or detrimental (nocebo group) effects of a dietary supplement. To our knowledge, the present study is the first on postural control to include both placebo and nocebo instructions in a single experimental design. By directly comparing placebo and nocebo effects with a group that received no-treatment (to control for repeated testing), we are able to demonstrate a bidirectional postural response to positive and negative verbal suggestions. Specifically, our data indicate that a nocebo procedure can negatively modulate objective and subjective postural stability compared to a control group. In contrast, our placebo group presented with a marked improvement in postural control and increased perceived stability following expectation manipulation. Our findings indicate that positive and negative performance expectations, evoked by instructional manipulation, can profoundly influence performance expectation and both objective and subjective postural stability. Postural control is clearly susceptible to expectation manipulation, which could have important practical implications and repercussions on training interventions and rehabilitation programs. For example, physical therapists or practitioners who are not fully aware of the power of words may inadvertently elicit placebo or nocebo effects, which may substantially modulate the efficacy of evidence-based interventions. There may also be important practical implications for practitioners in other sports-related professions such as coaches and teachers in the importance of psychological factors (i.e., expectations) in shaping performance.

### Performance expectations, belief manipulation and perceived change in performance

We initially hypothesized that administration of a placebo procedure would induce enhanced expectations about balance performance. We anticipated the opposite effects for the nocebo procedure. Consistent with our hypothesis and previous literature (Winkler and Hermann, 2019), the placebo group showed a significantly higher performance expectation post-manipulation compared to the nocebo and control group (Figure 2). Similarly, the nocebo group showed a significantly lower performance expectation post-manipulation compared to the placebo and control group. These findings confirm that placebo and nocebo procedures were highly effective in inducing positive and negative performance expectations, respectively. We also found that participants in the placebo and nocebo group strongly believed that the treatment influenced their performance (Figure 3B) and believed the information they were given (Figure 3C), further supporting the credibility of our belief-manipulation procedures. Furthermore, as predicted, participants in the placebo group rated their perceived change in balance performance significantly better than the control group (*d* = 2.31). Similarly, participants in the nocebo group rated their perceived change in balance performance significantly poorer than the control group (*d* = 3.34). Taken together our findings clearly indicate that manipulation of performance expectations strongly affects the perceived change in balance performance and that participants strongly believed the efficacy of the treatments.

### Reduced postural sway following a placebo procedure

Reduced COP movements during quiet standing have previously been described in healthy young adults following a placebo procedure (Villa-Sánchez et al., 2019). In agreement with our hypothesis and previous literature, our findings confirm that participants in the placebo group exhibited a reduced range (greater precision achieved by the postural control system) of the COP and concomitantly increased perceived stability after expectation manipulation. Since postural sway in the control group did not decline, the smaller range of COP movements during quiet standing in the placebo group is unlikely to be caused by a learning effect. This raises an important question: what are the mechanisms that allow for a placebo procedure to reduce postural sway during quiet standing? There are at least two possibilities. One explanation could be related to an increased corticospinal excitability. There is strong evidence for a crucial contribution of several cortical structures (e.g., cerebral cortex) in maintaining upright stance (Jacobs and Horak, 2007; Bolton et al., 2012; Mierau et al., 2017). These brain structures could potentially be exploited by placebo procedures. For example, a previous study showed that a placebo procedure increased the activity of the primary motor cortex, increasing the excitability of the corticospinal system (Fiorio et al., 2014). Therefore, we speculate that the reduced postural sway reported here could be partly due to increased voluntary drive to the motor cortex, which would logically elicit favorable adaptations in balance performance.

Another possible important factor facilitating the placebo effects reported in the present study could be related to increased motivation (i.e., choice, effort and persistence) or self-efficacy (i.e., the belief that one can successfully execute a task in a specific context; Bandura et al., 1997) following expectation manipulation. For example, there is clear evidence showing that psychological factors such as motivation, self-efficacy, and attentional focus can impact balance performance (Wulf and Lewthwaite, 2009; Lewthwaite and Wulf, 2010; Wulf et al., 2012; Chua et al., 2020). Additionally, our placebo procedure may have enhanced positive affect and resulted in increased effort. However, in a previous study, positive verbal suggestions elicited a reduction in postural sway without any changes in balance effort (measured using the Borg 0–10 scale; Borg, 1982; Villa-Sánchez et al., 2019). Therefore, the relative contribution of increased effort and motivation on postural stability following a placebo procedure remain unclear. In the present study we did not assess balance effort or motivation because we assumed that all participants would approach balance testing with reasonable effort and would be motivated to do their best. Future research should measure motivation and effort to help elucidate the relative contribution of these factors on postural control in response to enhanced performance expectancies.

### Increased postural sway following a nocebo procedure

The most striking finding presented here was a pronounced increase in postural sway in the nocebo group compared to the control group. Of course, an interesting question concerns the mechanism by which the nocebo procedure resulted in changes in COP movements during quiet standing. From a neurobiological perspective, nocebo procedures can inhibit dopaminergic neurological systems (resulting in decreased motivation/effort and self-efficacy; Beedie et al., 2018; Horváth et al., 2021), which may in turn hinder balance performance. It is also plausible that reduced performance expectations impaired balance performance through individuals directing attentional focus internally, in an attempt to consciously control movement. For example, research has described how consciously processing movements leads to distorted perceptions of instability (Ellmers et al., 2021), in addition to increased postural stability (Chow et al., 2019). However, we did not measure conscious movement processing in the present study and are therefore unable to determine the extent to which negative performance expectancies influenced attentional focus and subsequent balance performance. This issue should be explored in future studies.

An interesting feature of the data presented here was that participants in the nocebo group presented with feelings of fatigue, weakness and drowsiness after expectation manipulation. In this study, we gave detailed information about the (sham) mechanisms and subsequent effects on balance performance and explicitly pointed out the potential adverse symptoms that may be experienced in response to the nocebo procedure. In agreement with previous studies (Winkler and Hermann, 2019), fatigue was described as a side effect in the nocebo group. Unique to the present investigation, participants in the nocebo group also reported increased levels of weakness and drowsiness. Anecdotally, several participants in the nocebo group reported to postural tasks feeling “harder to perform” after expectation manipulation. However, we did not directly measure the amount of effort that was required to maintain balance in this study. Future studies should assess balance effort to help elucidate the mechanisms responsible for the nocebo effect on balance performance reported here.

### Subjective postural stability

Another important finding from the present work is that performance expectations exert a strong influence over perceptions of balance performance (i.e., perceived stability), even when accounting for *actual* task performance (i.e., objective postural sway outcomes). Interestingly, this relationship was only present post-manipulation; with performance expectations failing to predict perceived stability pre-manipulation. These findings fit with Predictive Processing Frameworks of perception (e.g., Clark, 2015). These frameworks contend that perception is the consequence of an interaction between top-down expectations (“priors”) and bottom-up sensory input (“prediction errors”). The extent to which each of these ultimately influence perception is determined by their predicted reliability (“precision”). For instance, if walking along a familiar street when it is dark and visibility is poor, incoming sensory input would lack precision, and perception would therefore be more strongly influenced by prior knowledge of what one would *expect* to see. Highly precise expectations are argued to explain visual illusions and hallucinations, whereby the objective sensory input does not match the subjective perception (Clark, 2015). We similarly contend that the false feedback that participants received in the present study was viewed as a highly precise source of information. This had the effect of disproportionately influencing perceptions of instability and balance performance. Participants who expected that they would perform poorly perceived themselves to be more unstable than those who expected that they would perform well—irrespective of *actual* performance (i.e., objective postural stability). Given that numerous clinical balance disorders are characterized by distorted perceptions of instability [whereby actual and perceived stability are decoupled (Ellmers et al., 2021)], these novel findings have high levels of applied relevance. Future work should explore if such distortions of perceived instability can be altered through targeting faulty expectancies about balance performance.

### Implications

There are several important implications to be gleaned from the present study. We provide the first direct evidence that negative verbal suggestions can elicit marked increases in postural sway and reductions in perceived stability in healthy young adults. These findings are disconcerting and point towards negative suggestions and expectancies potentially interfering or even preventing balance adaptations during chronic training. Physical therapists, researchers, coaches, teachers, and practitioners who are not fully aware of the power of words may unknowingly and unintendedly introduce negative expectations that could have detrimental repercussions on testing and training. Although we manipulated performance expectations by asking participants to ingest a pill, even subtle expectancy manipulation, such as providing positive and negative performance feedback, can negatively influence balance performance (Wulf and Lewthwaite, 2009; Lewthwaite and Wulf, 2010; Wulf et al., 2012; Chua et al., 2020). Although participants should clearly be informed about potential risks associated with training (i.e., exercise-induced falls), care should be taken to avoid inadvertently eliciting iatrogeny (see Evers et al., 2021 for expert consensus).

Contrary to negative expectations, placebo effects facilitate optimal balance performance. The practical significance of these findings, as evidenced by the large effect sizes, appear to be quite meaningful. From an applied perspective, these findings may suggest that placebo procedures could be exploited in physical training to improve balance performance, and could also be used to increase beliefs/expectations about balance performance in those older adults with disproportionately low levels of balance confidence (e.g., Delbaere et al., 2010). On one hand, overselling the placebo effect might prove ethically problematical; trust between client and health professionals, or between participant and scientist, should be paramount (Beedie and Foad, 2009). Instead, through the use of positive verbal suggestions about the efficacy of an intervention, practitioners can use the information presented here to increase the likelihood of performance improvements (Evers et al., 2021). For example, the physical therapist might provide the following disclosure to their patient: “*I recommend we add volitional step training in your program. It has been shown that this type of training can be highly effective in reducing the risk of falls. The use of volitional step training is very beneficial for your training and performance*”. Such information is honest, evidence based (see Okubo et al., 2017) and aims to engender a positive belief in the effectiveness of the treatment. Another important implication is that expectations appear to be relatively easy to manipulate and are therefore an effective target to induce changes in balance outcomes. From a theoretical standpoint, Self-Efficacy Theory suggests that social persuasion (most commonly verbal persuasion) can enhance self-efficacy, as long as it comes from a reputable source (Bandura et al., 1997). Collectively, there are considerations pertaining to the delivery and potential manipulation of beliefs/expectations that must be taken into account.

### Limitations

This study provides a novel contribution to the literature, and a solid platform for future investigations to examine the effects of positive and negative expectancies on postural control. However, the findings of the present study should be interpreted in light of the study limitations. Firstly, our study was limited to healthy young adults. There is a reasonable basis for expectation that verbal suggestions might be even more potent in older adults and clinical patients. Thus, more studies are warranted to provide a more definitive view of placebo and nocebo effects on postural stability, controlling for age, sex and inclusion of more diverse groups. Secondly, the quiet standing balance task used here may not adequately stress the postural control system and represent a relatively small subset of our balance repertoire (Hill et al., 2020). Although we increased the level of difficulty of balance tasks by manipulation of stance (bipedal vs. unipedal), we did not measure other components of balance, including dynamic steady-state (i.e., walking), proactive (i.e., reaching), and reactive (i.e., responding to an unpredicted perturbation, such as slip or trip) abilities. Therefore, future studies should seek to use a more comprehensive battery of balance assessments to determine which balance functions are susceptible to positive and negative verbal suggestions. Although, the 36-item GASE has good internal consistency (Cronbach’s *α* = 0.89; Rief et al., 2011), we only presented participants with the symptoms that we thought would occur relatively quickly after the pills were consumed. Therefore, we are unable to confirm the validity and reliability of the GASE as used here. Finally, the mechanistic basis of the increase (nocebo) and decrease (placebo) in postural sway remains to be elusive (and likely complicated). Neurophysiological investigations are needed to elucidate potential mechanisms underpinning the placebo- and nocebo-induced changes in balance. Additional examination of task specific anxiety, balance confidence, effort and motivation would also be quite valuable given their relevance to postural control.

## Conclusion

The present investigation represents the first study to directly compare the influence of placebo and nocebo instructions on objective and subjective postural stability. To summarize, the present study shows that opposite verbal suggestions (i.e., placebo vs. nocebo) result in distinct postural outcomes. Specifically, placebo procedures result in a pronounced reduction in postural sway and increase in subjective ratings of postural stability, whilst nocebo procedures elicited a marked increase in postural sway and reduction in subjective stability. Positive and negative expectancies are a double-edged sword for postural control, and clinicians should consider how their instructions and feedback during therapeutic intervention may influence patients’ expectation judgments.

## Data Availability Statement

The raw data supporting the conclusions of this article will be made available by the authors, without undue reservation.

## Ethics Statement

The studies involving human participants were reviewed and approved by Coventry University Ethics Committee. The patients/participants provided their written informed consent to participate in this study.

## Author Contributions

MH conceived and designed research. MH and KR conducted experiments. MH performed the analyses and wrote the manuscript. MH, AM, MP, MD, and TE revised the manuscript. All authors contributed to the article and approved the submitted version.

## Funding

We acknowledge support by the Open Access Publication Fund of the Centre for Sport, Exercise and Life Science, Coventry University.

## Footnotes

^1^ www.randomizer.org
